# Thymus size and its correlates among children admitted with severe acute malnutrition: a cross-sectional study in Uganda

**DOI:** 10.1186/s12887-020-02457-3

**Published:** 2021-01-04

**Authors:** Nicolette Nabukeera-Barungi, Betty Lanyero, Benedikte Grenov, Henrik Friis, Hanifa Namusoke, Ezekiel Mupere, Kim F. Michaelsen, Christian Mølgaard, Maria Wiese, Dennis S. Nielsen, Musemma K. Mohammed, Vibeke B. Christensen, Maren Rytter

**Affiliations:** 1 Department of Paediatrics and Child Health, Makerere University College of Health Sciences, P.O. Box 7072, Kampala, Uganda; 2 Department of Nutrition, Exercise and Sports, University of Copenhagen, DK-1958 Frederiksberg C, Denmark; 3 Mwanamugimu Nutrition Unit, Department of Paediatrics, Mulago National Referral Hospital, P.O. Box 7051, Kampala, Uganda; 4 Department of Paediatrics and Adolescent Medicine, Rigshospitalet, DK-2100 Copenhagen Ø, Denmark; 5 Department of Food Science, University of Copenhagen, DK-1958 Frederiksberg C, Denmark

**Keywords:** Thymus, Ultrasound, Size, Severe acute malnutrition, Breastfeeding, Children

## Abstract

**Background:**

Malnutrition continues to be a major cause of mortality and morbidity among children in resource limited settings. Children with severe acute malnutrition (SAM) experience severe thymus atrophy, possibly reflecting poor immune function. This immune dysfunction is responsible for the severe infections they experience which lead to mortality. Since their immune dysfunction is not fully understood and there has been a lapse in research in this field, more research is needed. Knowing the correlates of thymus size may help clinicians identify those with more severe atrophy who might have more severe immune impairment. We aimed to describe thymus size and its correlates at admission among children hospitalized with SAM.

**Methods:**

This cross-sectional study involved children 6-59 months admitted with complicated SAM in Mulago National Referral Hospital. Well-nourished children from same communities were used as a community reference group for thymus size. At admission, thymus size was measured by ultrasound scan. Demographic, clinical and laboratory variables were identified at admission. A linear regression model was used to determine correlates of thymus size among children with SAM.

**Results:**

Among 388 children with SAM, the mean age was 17±8.5 months and 58% were boys. The mean thymus size was 3.14 (95% CI 2.9; 3.4) cm^2^ lower than that of the 27 healthy community reference children (1.06 vs 4.2 cm^2^, *p*<0.001) when controlled for age. Thymus size positively correlated with current breastfeeding (0.14, 95% CI 0.01, 0.26), anthropometric measurements at admission (weight, length, mid-upper-arm circumference, weight-for-height Z scores and length-for-age Z scores) and suspected tuberculosis (0.12, 95% CI 0.01; 0.22). Thymus size negatively correlated with > 2 weeks duration of sickness (-0.10; 95% CI -0.19; -0.01).

**Conclusion:**

The thymus is indeed a barometer for nutrition since all anthropometric measurements and breastfeeding were associated with bigger thymus. The immune benefits of breastfeeding among children with SAM is underscored. Children with longer duration of illness had a smaller thymus gland indicating that infections have a role in the cause or consequence of thymus atrophy.

## Background

Malnutrition continues to be a major cause of morbidity and mortality among children, with 45% of all child deaths being estimated to be linked to malnutrition [1]. The high mortality rate among children hospitalized with severe acute malnutrition (SAM) is largely due to the high burden of infections they experience, with diarrhoea and pneumonia being the most common [2, 3]. This increased susceptibility to infection is not well understood but it is likely due to changes in immune functions associated with malnutrition [4]. Despite gaps in understanding immune functions among children with SAM, there has been a lapse in research in this area in the last 10 years [4], highlighting the need for further research.

The thymus gland is a lymphoid organ where maturation of the bone marrow derived T lymphocyte takes place. Children with SAM have been found to have severe atrophy of the thymus gland [3–6]. This could be as a result of several micronutrient deficiencies [7, 9], hormonal changes [10, 11], lack of energy and building blocks [12] and concurrent infections [13].

The thymus gland has been found to be sensitive to environmental factors, and its size seems to reflect the influence of these different factors [14, 15]. Since most studies on factors affecting thymus size have involved healthy children, a knowledge gap still exists for the children with SAM who suffer severe thymus atrophy. Knowing the correlates may help clinicians identify those with more severe atrophy who might have more severe immune impairment. In addition, it may contribute to understanding the immune functions among children with SAM. We therefore set out to determine the thymus size and its correlates at admission among children who were admitted with SAM in Mulago Hospital.

## Methods

### Study design and setting

This was a cross-sectional study which was part of a randomized controlled trial assessing the effect of probiotics on diarrhea among children admitted with SAM; registered at www.isrctn.com (ISRCTN16454889) and whose findings were published [16]. Patients were enrolled over a period of 18 months starting March 2014. The study was conducted at the Mwanamugimu Nutrition Unit (MNU) located in Mulago National Referral Hospital, a large teaching hospital with a 1500 bed capacity in Kampala, Uganda. The unit admits about 120 severely malnourished children per month for inpatient therapeutic care and also provides outpatient nutritional rehabilitation at the MNU Outpatient Therapeutic Care (OTC) clinic.

### Selection criteria

We included children aged 6–59 months admitted with SAM in MNU and whose caretakers consented to participate in the study. SAM was defined as mid-upper arm circumference (MUAC) < 11.5 cm or weight-for-height Z score (WHZ) <-3 SD or bipedal pitting edema. As part of the randomized controlled trial, we excluded patients with severe conditions such as shock, severe respiratory distress at admission, weight below 4.0 kg and obvious disability whose response to nutritional rehabilitation is usually compromised. We also included 30 apparently healthy children from similar social status, age group and communities as study patients with SAM but whose WHZ were >-1. These were selected to provide the reference thymus size for non-malnourished children in the same setting.

### Data collection

Children who fulfilled the eligibility criteria during the study period were consecutively enrolled until the required sample size was reached. After providing written informed consent, a questionnaire was administered by a study pediatrician or medical officer at enrollment. The questionnaire included medical history focusing on demographics, description of presenting symptoms, physical examination and diagnosis. More detailed dietary history and socioeconomic history was taken the following day. As part of the physical examination, anthropometric measurements were taken by nutritionists three times and the average was taken. Length or height was measured using an infant length board (Infant/Child Shorr-Board® Maryland, USA) and MUAC using a measuring tape, both to the nearest 1 mm. Body weight was measured using a digital scale (Seca 813 Hamburg, Germany) to the nearest 0.1 kg. The WHZ were calculated using the Child Growth Standards of the World Health Organization (WHO) [17]. The healthy controls were subjected to the same questionnaires and tests as the children with SAM. Treatment of SAM children was instituted according to the Integrated Management of Acute Malnutrition (IMAM) guidelines [18] but transition of feeds was according to the WHO guidelines [19].

### Blood tests

Four ml of blood were drawn from venipunctures into heparinized vacutainer tubes (Becton Dickinson, Franklin lakes, NJ USA). Samples for complete blood count were analyzed using a Coulter counter and expressed as cells per cubic millimeter (cells/mm^3^). HIV serological testing was done using rapid tests (Determine HIV-1/2 from Abbott Laboratories, USA), and positive samples were confirmed with HIV 1/2 Stat-Pak Dipstick Assay kit (Chembio Diagnostic systems, INC, USA). All children below 18 months who had a positive serology test were sent for an HIV DNA/PCR test done at the hospital’s HIV clinic. Plasma was obtained by centrifuging at 1300–2200 G for 10 minutes then stored at -80^o^C until shipped on dry ice to the University of Copenhagen, Department of Nutrition, Exercise and Sports, Denmark. Plasma C-reactive protein (CRP) was measured by high sensitive kit on an ABX Pentra 400 (Horiba, Montpellier, France).

### Thymus gland measurement

The dependent variable was thymus size, measured by ultrasound scan. Ultrasound scan of the thymus gland was done on the second day of admission by one of the two pediatricians on the study who were trained before the study began.

**Fig. 1:**
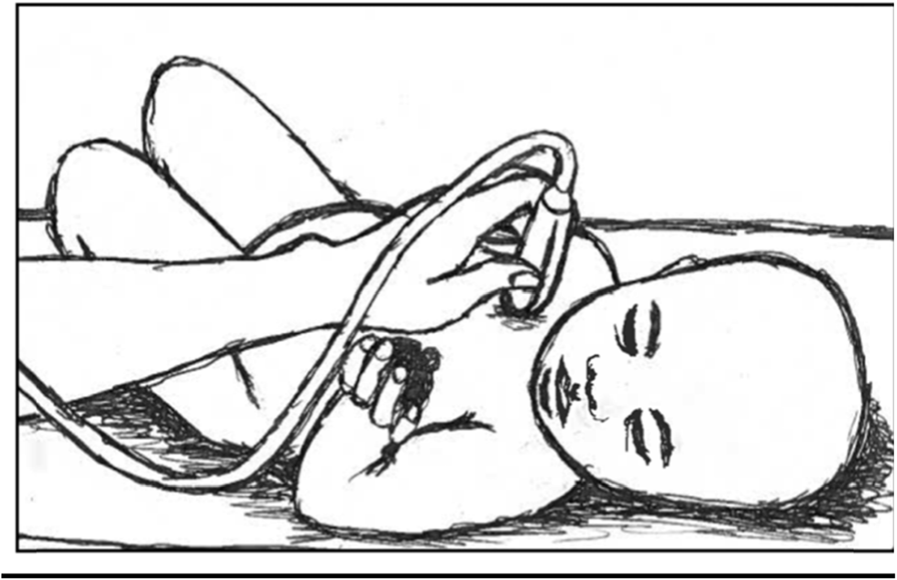
Position for ultrasound scan (original picture)

Scans took place at the MNU ward in a well screened-off bed to provide darkness for better visibility. Attempts were made to keep the children calm during the procedure. An ultrasound scanner (SonoScape A6 Guangdong, China) with a pediatric abdominal probe (curved) was used. The procedure was done with the child lying on the back or in the mother’s lap as shown in Fig. 1.

**Fig. 2:**
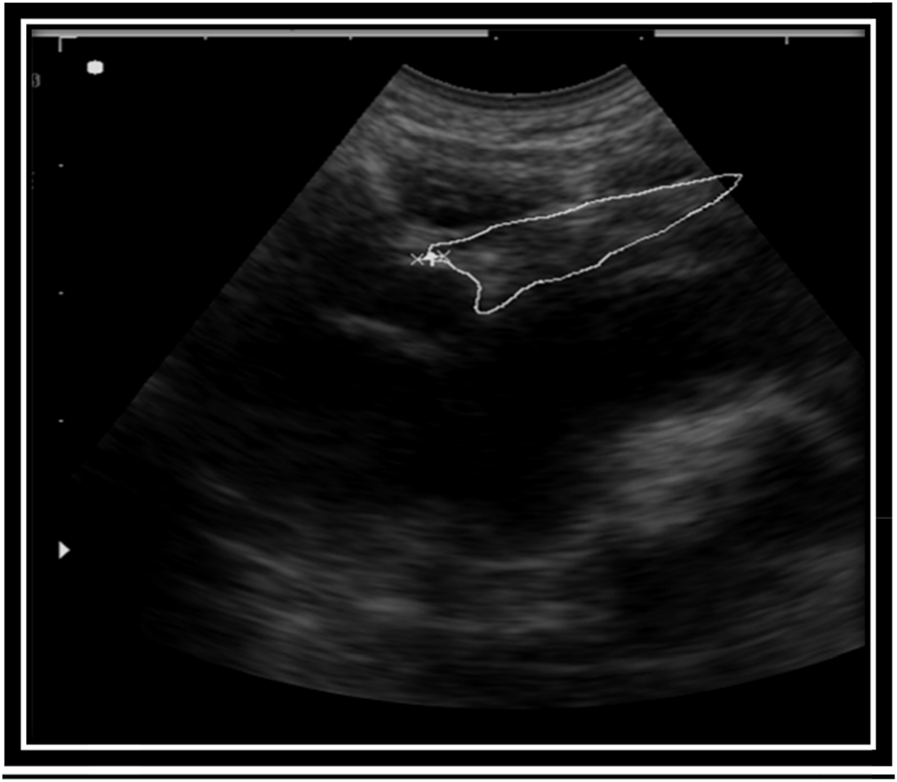
Ultrasound scan of a child with SAM

The sagittal area was obtained by placing the transducer at 90 degrees to the upper sternum where the thymus was visualized as having a homogeneous echogenicity, similar to that of the liver and spleen as shown in Fig. 2. The circumference of the largest lobe of the thymus was tracked and the area measurement recorded. All measurements were done in duplicate, and the average of the two measurements used. One investigator (NNB) carried out about 96% of the scans.

Sample size of 400 was calculated for the RCT from which this study was derived.

### Data analysis

Data was double-entered in EpiData v.3.1 (EpiData, Odense, Denmark) and analyzed using STATA version 2 (College Station, TX, USA). Means and medians were calculated for continuous variables. Cross tabulations were made and the Chi Square test was used for the categorical variables and t-test used to compare means between children with SAM and healthy controls. One child with SAM had an abnormally large thymus of 5.11 cm^2^ and was removed from the analysis because he was suspected to have a lymphoma. Three children who had an invisible thymus were allocated half of the smallest visible thymus size and included in the analysis. The 11 children with SAM and 3 community reference children who missed having an Ultrasound scan were eliminated from the analysis. A linear regression model was used to determine factors associated with thymus size among SAM children. Univariate analysis for correlates of thymus size was done first, then each variable was controlled for age and sex. Those with *p*-value < 0.05 were considered to be statistically significant.

## Results

Among 400 children with SAM, the mean age was 17 ± 8.5 months and 58% were boys (Table 1). The 30 healthy controls were older than the children with SAM (*P* = 0.001), and more likely to be breastfed (*P* = 0.001). Oedematous SAM was present in 66% (*N* = 263) of the children with SAM (Table 2), and 11% (*N* = 43) were confirmed to be HIV infected.

**Table 1:** Socio-demographic and anthropometric characteristics of 400 SAM children and 30 healthy controls

	SAM^1^ *N* = 400	**Healthy controls**^1^, *N* = 30	**p**	
Male sex	230 (58)	18 (60)	0.78	
Age, months	17 (± 8.5)	27 (± 13.5 )	< 0.001	
Anthropometry				
Mid-upper-arm circumference, cm	11.6 (± 1.5)	15.2 (± 1.3)	< 0.001	
Weight-for-height/length Z-scores	-2.6 (± 1.5)	0.6 (± 1.1)	< 0.001	
Height/length-for-age Z-scores	-3.1 (± 1.4)	-1.2 (± 1.8)	< 0.001	
Birth weight, kg	3.3 (± 0.82)	3.6 (± 0.7)	0.23	
Breastfed				
Currently	54 (14)	12 (40)	0.001	
Maternal factors				
Lives with child	317 (81)	25 (83)	0.26	
Education ^a^	211(54)	12 (40)	0.30	
Married/cohabiting	212 (55)	23 (77)	0.01	
HIV positive	109 (34)	2 (6)	< 0.001	

^1^Presented as frequencies (%) or as mean (± standard deviation) For some categorical variables, numbers do not add up due to missing data. ^a^ Mother’s education is ≤ completed primary school

Thymus scans were done in 389 (97%) children with SAM and 27 controls. Almost all children with SAM [386 (99%)] and all the healthy controls were found to have visible thymus. The mean thymus gland size among 388 children with SAM was 1.06 (± 0.41) cm^2^ compared to 4.20 (± 0.93) cm^2^ among healthy controls. After adjustment for age, the mean thymus size was 3.14 (2.9; 3.4) cm^2^ lower in SAM children (*P* < 0.0001).

**Table 2:** Clinical and laboratory characteristics of 400 SAM children

	**N**^**a**^	
Clinical presentation		
History		
Sick for > 2 weeks	260 (65)	
Severity of sickness VAS ^b^	6 (± 2)	
Fever	211 (53)	
Cough	262 (66)	
Examination		
Oedema	263 (66)	
Oral thrush	84 (21)	
Dermatosis	27 (7)	
Diagnosis		
Diarrhoea	244 (61)	
Pneumonia	68 (17)	
Suspected tuberculosis	76 (19)	
Suspected septicaemia	96 (24)	
HIV status		
Positive	43 (11)	
Exposed, negative	72 (18)	
Unexposed, Negative	252 (63)	
Blood tests		
C-Reactive Protein, mg/l *(N = 352)*		
< 5	92 (26)	
5–15	80 (23)	
> 15	180 (51)	
Leucocytes, x10^9^/L *(N = 298)*		
Total lymphocytes	6 (± 2.9)	
Total neutrophils	5 (± 5.0)	
Haemoglobin, g/dl (*N* = 298)	9 (± 2.2)	

^a^ Data presented as frequencies (%) or as mean (± standard deviation). For some categorical variables, numbers do not add up due to missing data.

^b^ Evaluated by the child’s caregiver on a visual analogue scale from 0 to 10

Correlates of thymus size are summarized in Table 3. Positive correlates included current breastfeeding [β 0.14, 95% CI (0.01, 0.26)], suspected tuberculosis at admission [β 0.12, 95% CI (0 0.01; 0.22)], all anthropometric measurements at admission, including MUAC [β 0.03, 95% CI (0.0003; 0.06)], WHZ [β 0.03, 95% CI (0.04; 0.06)], weight [β 0.06, 95% CI (0.02; 0.09)], length [β 0.02, 95% CI (0.004; 0.03)] and length-for-age Z scores [β 0.05, 95% CI (0.02; 0.08)]. On the other hand, sickness for 2 weeks or more appeared to be negatively correlated with thymus size [β -0.10; 95% CI (-0.19; -0.01)].

**Table 3:** Linear regression of correlates of thymus area at admission among 388 children with SAM

		Univariate regression		Age and sex adjusted regression	
	N ^a^	β (95% CI) ^b^	p	β (95% CI) ^b^	p
Male sex	388	0.01 (-0.07; 0.10)	0.79	0.01 (-0.07; 0.10)	0.78
Age, months	388	0.001(-0.004; 0.01)	0.76	0.001 (-0.004; 0.01)	0.75
Anthropometry at admission					
Weight, kg	382	0.03 (0.01; 0.06)	0.01	0.06 (0.02; 0.09)	0.001
Length, cm	379	0.01 (1.03; 0.01)	0.05	0.02 (0.004; 0.03)	0.01
MUAC, cm	382	0.03 (0.001; 0.06)	0.046	0.03 (0.0003; 0.06)	0.048
Weight-for-length, z-score	380	0.03 (0.01; 0.06)	0.02	0.03 (0.04; 0.06)	0.02
Length-for-age, z-score	380	0.04 (0.01; 0.07)	0.02	0.05 (0.02; 0.08)	0.003
Clinical history					
Severity of sickness VAS^c^	387	-0.01 (-0.03; 0.01)	0.49	− 0.01 (-0.03; 0.01)	0.47
Sick for > 2 weeks	377	-0.10 (-0.19; -0.01)	0.03	-0.10 (-0.19; -0.01)	0.03
Cough	387	0.02 (-0.06; 0.11)	0.60	0.02 (-0.06; 0.11)	0.60
Fever	387	-0.003 (-0.09; 0.08)	0.95	-0.003 (-0.09; 0.08)	0.95
Examination					
Oedema	388	-0.06 (-0.15; 0.03)	0.19	-0.06 (-0.15; 0.03)	0.16
Skin hypopigmentation	388	-0.04 (-0.13; 0.04)	0.34	-0.04( -0.13; 0.04)	0.33
Dermatosis	388	-0.15 (-0.31; 0.01)	0.07	-0.15 (-0.31; 0.01)	0.07
Hair depigmentation	383	-0.10 (-0.19; 0.001)	0.05	-0.10 (-0.20; 0.0002)	0.05
Oral thrush ^g^	387	0.01 (-0.09; 0.11)	0.80	0.01 (-0.09; 0.11)	0.80
Diagnosis					
Diarrhoea	387	-0.03 (-0.12; 0.05)	0.48	-0.03 (-0.12; 0.06)	0.50
Pneumonia	388	0.04 (-0.07; 0.22)	0.49	0.04 (-0.07; 0.15)	0.49
Suspected tuberculosis	388	0.12 (0.01; 0.2)	0.03	0.12 (0 0.01; 0.22)	0.03
Suspected septicaemia	386	0.08 (-0.02; 0.18)	0.12	0.08 (-0.02; 0.18)	0.12
Child HIV status	367				
Negative		-	-	-	-
Positive		-0.08 (-0.22; 0.05)	0.21	-0.08 (-0.22; 0.05)	0.22
Exposed, negative		-0.01 ( -0.12; 0.10)	0.80	-0.01 ( -0.12; 0.10)	0.80
C-reactive protein, mg/L	343				
< 5		-	-	-	-
5–15		-0.01 (-0.14, 0.11)	0.85	-0.01 (-0.14, 0.11)	0.85
> 15		-0.03 (-0.14, 0.08)	0.60	-0.03 ( -0.14, 0.08)	0.59
Leukocytes, X10^9^/L,					
Neutrophils > 9	293	0.13 (-0.02; 0.28)	0.08	0.13 (-0.02; 0.28)	0.08
Lymphocytes > 10	293	0 0.12 (-0.05; 0.30)	0.17	0.13 (-0.05; 0.30)	0.16
Haemoglobin, g/dl	292	-0.001 (-0.03; 0.02)	0.96	-0.001 (-0.03; 0.02)	0.97
Breastfed	384				
Previously		-	-	-	-
Currently		0.13 ( 0.01, 0.25)	0.04	0.14 ( 0.01, 0.26)	0.03
Never		-0.09 ( -0.37; 0.18)	0.50	-0.09 ( -0.37; 0.18)	0.50
Mother					
Lives with child	388	0.09 (-0.02; 0.19)	0.11	0.09 (-0.02; 0.19)	0.10
Education:	384				
Post primary		-	-	-	-
≤primary		0.06 (-0.03; 0.14)	0.19	0.05 (-0.03; 0.14)	0.20

N ^a^: number of observations included in analysis. β = beta coefficient ^b^ (95% confidence interval) and P = *p*-values. ^c^ Evaluated by the child’s caregiver on a visual analogue scale from 0 to 10

## Discussion

Our study found the mean thymus gland size among 388 children with SAM was 1.06 (± 0.41) cm^2^ compared to 4.20 (± 0.93) cm^2^ among healthy controls. The factors which were positively associated with thymus size were current breastfeeding, suspected tuberculosis at admission and all anthropometric measurements at admission while sickness for 2 weeks or more appeared to be negatively correlated with thymus size The children with SAM had severe thymus atrophy as has been reported by several studies involving this population [5–8]. Our measurements were similar to another study which found a mean thymus area of 1.3 cm^2^ in SAM, and 3.5 cm^2^ in healthy children [5]. The slight difference in these studies can be explained by inter-observer differences, which make it difficult to compare sizes. Although thymus size is usually measured using thymic index (multiplying the transverse diameter by the sagittal area), thymus area is also an acceptable method and has been used in other studies involving children with SAM [5–7].

The mechanism of thymus atrophy in SAM is not fully understood. Animal studies suggest that hormonal factors play a role. Thymus growth and function is regulated by several hormones including Leptin, Prolactin and Growth hormone, mediated by insulin-like growth factor 1, all of which are low in malnutrition [20]. In addition, zinc deficiency which is rampant in SAM has been suggested to contribute to thymic atrophy [4]. Infections have also been found to contribute to thymic atrophy [21], but it is not clear whether they are the cause or consequence.

Children who were breastfed at the time of study had a larger thymus when controlled for age. Some other studies involving children, who are not malnourished also report that breastfeeding children have a bigger thymus [14, 22, 23]. Cytokines like IL-7 present in breast milk are vital for development of the thymus and hence age related thymic atrophy is associated with low IL-7 [24]. It has been hypothesized that the increased IL-7 in their mothers’ milk could be responsible for the bigger thymus since the thymus was bigger in infants whose mothers had higher IL-7 in their milk during the harvest season in Gambia [25]. In addition, all anthropometric measurements were positively correlated to thymus area. This is similar to other studies among non-malnourished children [14, 24] and SAM children [5]. Children who had been sick for ≥ 2 weeks had a smaller thymus. The delay between disease onset and thymus atrophy likely reflects that thymus atrophy takes time to develop. Another post-mortem study among 234 fetuses and young children similarly found that among prenatal patients, thymus weight was related to the duration of acute illness [26], and thymus size has been used to determine duration of illness by pathologists. Children with suspected tuberculosis at admission had a larger thymus in our study. This is an unexpected finding, and its significance remains unclear. Literature suggests that enlarged mediastinal lymph nodes could not be easily distinguished from the thymus [27], and this could have been the case in our study. Although HIV is known to cause thymus atrophy by direct destruction of thymocytes [28], it was not associated with thymus size in our study. This was unexpected since only 14/43 were receiving antiretroviral treatment at admission.

The main strength of our study is that we had large numbers of well-characterized children with SAM and the thymus scans were almost exclusively performed by one trained investigator (NNB). One limitation is that we used clinical diagnosis of suspected tuberculosis and septicemia, which were subjective methods and yet the former was a positive correlate. Furthermore, since the study was observational, we are unable to establish causality. It remains unclear whether thymus atrophy is a cause or effect of malnutrition. This calls for a prospective study looking at children who are at risk of malnutrition. Considering the large sample size, these results are generalizable to children with SAM in similar settings. Future research should look at how thymus size predicts function using T cell receptor excision cycles.

## Conclusions

In conclusion, our study found that children with SAM had severe thymic atrophy. Children with higher anthropometric measurements and those who were breastfed had a bigger thymus. Breastfeeding should continue to be promoted since it may have current immune-modulating effects even among children with SAM.

## Data Availability

Data will be made available by the corresponding authors upon reasonable request, and with the approval of all bodies from whom permissions are required.

## Abbreviations

CBC: Complete Blood Counts
CRP: C-Reactive protein
DBS: Dried blood spots
HIV: Human Immunodeficiency Virus
IMAM: Integrated Management of Acute Malnutrition
ITC: Inpatient Therapeutic Care
MNU: Mwanamugimu Nutrition Unit
MUAC: Mid-upper arm circumference
OTC: Outpatient Therapeutic Care
SAM: Severe Acute Malnutrition
WHO: World Health Organization
WHZ: weight-for-height Z score

## References

[1] Black RE, Victora CG, Walker SP (2013). Maternal and child undernutrition and overweight in low-income and middle-income countries. Lancet.

[2] Munthali T, Jacobs C, Sitali L, Dambe R, Michelo C (2015). Mortality and morbidity patterns in under-five children with severe acute malnutrition (SAM) in Zambia: a five-year retrospective review of hospital-based records (2009–2013). Archives of Public Health..

[3] Liu L, Johnson HL, Cousens S, Perin J, Scott S, Lawn JE, Rudan I, Campbell H, Cibulskis R, Li M, Mathers C, Black RE, for the Child Health Epidemiology Reference Group of WHO and UNICEF (2012). Global, regional, and national causes of child mortality: an updated systematic analysis for 2010 with time trends since 2000. Lancet.

[4] Rytter MJH, Kolte L, Briend A, Friis H, Christensen VB (2014). The immune system in children with malnutrition–a systematic review. PloS One.

[5] Rytter MJH, Namusoke H, Ritz C, Michaelsen KF, Briend A, Friis H, et al. Correlates of thymus size and changes during treatment of children with severe acute malnutrition: a cohort study. BMC Pediatr. 2017;14(1):70. 17. 10.1186/s12887-017-0821-0 PMC5348758 28288591

[6] Chevalier P, Sevilla R, Sejas E, Zalles L, Belmonte G, Parent G (1998). Immune recovery of malnourished children takes longer than nutritional recovery: implications for treatment and discharge..

[7] Parent G, Chevalier P, Zalles L (1994). In vitro lymphocyte-differentiating effects of thymulin (Zn-FTS) on lymphocyte subpopulations of severely malnourished children. Am J Clin Nutr.

[8] Nassar MF, Younis NT, Tohamy AG, Dalam DM, El Badawy MA. T-lymphocyte subsets and thymic size in malnourished infants in Egypt: a hospital-based study. East Mediterr Health J Rev Santé Méditerranée Orient Al-Majallah Al-Ṣiḥḥīyah Li-Sharq Al-Mutawassiṭ. 2007;13(5):1031–42. 10.26719/2007.13.5.1031 18290395

[9] Golden MH, Jackson AA, Golden BE. Effect of zinc on thymus of recently malnourished children. Lancet Lond Engl. 1977;19(8047):1057–9. 10.1016/s0140-6736(77)91888-8 72960

[10] De Mello-Coelho V, Savino W, Postel-Vinay MC, Dardenne M. Role of prolactin and growth hormone on thymus physiology. Dev Immunol 1998; 1998;6(3–4):317–323. 10.1155/1998/89782 PMC2276021 9814605

[11] Haeryfar SM, Berczi I (2001). The thymus and the acute phase response. Cell Mol Biol Noisy–Gd Fr.

[12] McMurray DN (1984). Cell-mediated immunity in nutritional deficiency. Prog Food Nutr Sci.

[13] Savino W, Dardenne M, Velloso LA, Dayse Silva-Barbosa S. The thymus is a common target in malnutrition and infection. Br J Nutr. 2007;98(Suppl 1):11–6. 10.1017/S0007114507832880 17922946

[14] Moore SE, Prentice AM, Wagatsuma Y, Fulford AJC, Collinson AC, Raqib R, et al. Early-life nutritional and environmental determinants of thymic size in infants born in rural Bangladesh. Acta Paediatr Oslo Nor 1992. 2009;98(7):1168–75. 10.1111/j.1651-2227.2009.01292.x PMC2721967 19432828

[15] Collinson AC, Moore SE, Prentice AM (2003). Birth season and environmental influences on patterns of thymic growth in rural Gambian infants. Acta Paediatr.

[16] Grenov B, Namusoke H, Lanyero B, Nabukeera-Barungi N, Ritz C, Mølgaard C, et al. Effect of Probiotics on Diarrhea in Children With Severe Acute Malnutrition: A Randomized Controlled Study in Uganda. J Pediatr Gastroenterol Nutr. 2017;64(3):396–403. 10.1097/MPG.0000000000001515 28079729

[17] World Health Organization. The WHO Child Growth Standards [Internet]. WHO; 2014. Available from: http://www.who.int/childgrowth/en/.

[18] Ministry of Health. The Republic of Uganda. Integrated Management of Acute Malnutrition Guidelines [Internet]. 2010. Available from: http://www.unicef.org/uganda/IMAM_Guidelines_final_version.pdf.

[19] World Health Organization. Guideline: Updates on the management of severe acute malnutrition in infants and children. [Internet]. Geneva: World Health Organization; 2013. Available from: www.who.int/nutrition/.../guidelines/updates_management_SAM_infantandchildren/. 24649519

[20] Bartz S, Mody A, Hornik C, Bain J, Muehlbauer M, Kiyimba T (2014). Severe Acute Malnutrition in Childhood: Hormonal and Metabolic Status at Presentation, Response to Treatment, and Predictors of Mortality. J Clin Endocrinol Metab..

[21] Savino W, Dardenne M. Nutritional imbalances and infections affect the thymus: consequences on T-cell-mediated immune responses. Proc Nutr Soc. 2010;69(4):636–43. 10.1017/S0029665110002545 20860857

[22] Jeppesen DL, Hasselbalch H, Lisse IM, Ersbøll AK, Engelmann MDM. T-lymphocyte subsets, thymic size and breastfeeding in infancy. Pediatr Allergy Immunol Off Publ Eur Soc Pediatr Allergy Immunol. 2004;15(2):127–32. 10.1111/j.1399-3038.2004.00032.x 15059188

[23] Hasselbalch H, Jeppesen DL, Engelmann MD, Michaelsen KF, Nielsen MB. Decreased thymus size in formula-fed infants compared with breastfed infants. Acta Paediatr. 1996;85(9):1029–32. 10.1111/j.1651-2227.1996.tb14211.x 8888912

[24] Garly M-L, Trautner SL, Marx C, Danebod K, Nielsen J, Ravn H, et al. Thymus size at 6 months of age and subsequent child mortality. J Pediatr. 2008;153(5):683–8. 688.e1-3. 10.1016/j.jpeds.2008.04.069 18589444

[25] Ngom PT, Collinson AC, Pido-Lopez J, Henson SM, Prentice AM, Aspinall R. Improved thymic function in exclusively breastfed infants is associated with higher interleukin 7 concentrations in their mothers’ breast milk. Am J Clin Nutr. 2004 Sep;80(3):722–8. 10.1093/ajcn/80.3.722 15321814

[26] van Baarlen J, Schuurman HJ, Huber J. Acute thymus involution in infancy and childhood: a reliable marker for duration of acute illness. Hum Pathol. 1988;19(10):1155–60. 10.1016/s0046-8177(88)80146-1 3169723

[27] Nishino M, Ashiku SK, Kocher ON, Thurer RL, Boiselle PM, Hatabu H (2006). The Thymus: A Comprehensive Review. RadioGraphics..

[28] Vigano’ A, Vella S, Principi N, Bricalli D, Sala N, Salvaggio A, et al. Thymus volume correlates with the progression of vertical HIV infection. AIDS Lond Engl. 1999;13(5):F29-34. 10.1097/00002030-199904010-00001 10203377

